# A focus group study of women’s views and experiences of maternity care as delivered collaboratively by midwives and health visitors in England

**DOI:** 10.1186/s12884-018-2127-0

**Published:** 2018-12-27

**Authors:** Maria Raisa Jessica V. Aquino, Ellinor K. Olander, Rosamund M. Bryar

**Affiliations:** 1 Primary Care Unit, Department of Public Health and Primary Care, University of Cambridge, Cambridge, UK; 2 Centre for Maternal and Child Health Research, School of Health Sciences, City, University of London, London, UK

**Keywords:** Maternal health, Interprofessional collaboration, Women’s experiences, Qualitative enquiry, Thematic analysis, Pregnancy, Postnatal, Midwife, Health visitor

## Abstract

**Background:**

Research suggests that collaboratively delivered maternity care can positively impact health outcomes. However, women’s perspectives on models of care involving interprofessional collaboration between midwives and health visitors are not well understood. Accounts of women’s maternity care experiences are key to improving maternity services. This study considered women’s views and experiences of maternity care as collaboratively provided by midwives and health visitors in England.

**Methods:**

A qualitative focus group study with an exercise exploring women’s ideal maternity care pathway was conducted. Three focus groups were conducted in London, England between June and August 2017 with women who had had a child within 18 months prior to the study. The participants (*n* = 12) were recruited from two Children’s Centres in London, England. Data were analysed using thematic analysis.

**Results:**

Four themes were identified: ‘Women’s experiences of maternity care from midwives and health visitors’, ‘Midwife-health visitor communication’, ‘Midwife-health visitor collaboration for tailored care’, and ‘Women’s ideal maternity care pathway’. Regarding women’s experiences of interprofessional collaboration between midwives and health visitors, this was rarely encountered, but welcomed by women. Women’s observations of limited tailored care and co-ordination led to several suggestions to improve maternity care, including secure, shared medical recordkeeping systems, clarity on midwives’ and health visitors’ roles, as well as increased communication.

**Conclusions:**

Maternity care that is collaboratively delivered by midwives and health visitors, from the perspectives of the women in this study, is not routinely provided. However, women recognise the potential benefits of midwife-health visitor collaboration. Future research should explore service configurations that support integrated maternity care pathways, and evaluate the impact of midwife-health visitor collaboration on health and service outcomes.

**Electronic supplementary material:**

The online version of this article (10.1186/s12884-018-2127-0) contains supplementary material, which is available to authorized users.

## Background

Interprofessional collaboration is widely promoted across health services, including maternal and child health services, both in the UK [1, 2] and internationally [3]. According to the World Health Organization, interprofessional collaboration occurs when different healthcare professionals work together to improve care [4]. The mounting evidence concerning the importance of one’s early childhood to the rest of the lifespan puts interprofessional collaboration high on government agendas as a strategy for addressing women’s and their families’ unmet needs and improving outcomes [3, 5–7].

In the UK, midwives and health visitors (specialist community public health nurses) are key maternity care providers. These groups share overlapping professional remits both antenatally and postnatally and are encouraged to work together [8]. Specifically, Public Health England and Department of Health (UK) partnership pathway outlines this working relationship such that midwives and health visitors should be communicating with each other during and after pregnancy regarding the health and wellbeing of mother and baby [8]. Our recent systematic review of the international evidence on interprofessional collaboration between midwives and health visitors showed that collaboration in practice varied, and is influenced by interlinked structural (e.g. geographical distance, limited resources) and individual factors (e.g. communication, support for colleagues) [9].

Previous research suggests that collaborative maternity care models can have a positive impact on health outcomes [10], including breastfeeding [7], mental health and smoking cessation [11]. Conversely, poor interprofessional collaboration is associated with negative maternity care experiences, and can result in failures in care [12]. It is therefore important to identify women’s experiences of midwife-health visitor collaboration, and explore how they envisage maternity services to be developed.

Women’s involvement in the exploration of interprofessional collaboration care models in maternity is limited (e.g. [13, 14]), despite being service users [15]. Sandall and colleagues [12] have suggested that further research on women’s experiences of continuity of care models, which include various health professionals working together, is needed. A recent systematic review of continuity of care with doctors demonstrated that greater continuity of care (defined as repeated contact between a patient and a doctor) was associated with lower mortality [16]. Continuity of care is also encouraged in maternity care guidance set out by the National Health Service (NHS) in England [17].

Critical realism [18–20] is a philosophical approach that allows for the understanding of the layers shaping individuals’ experience and reality, and the links between these [21]. This asserts that reality is comprised of three levels: the empirical, the actual, and the real [19, 20]. In the context of midwife-health visitor collaboration, the *empirical* level concerns the directly observable, perceived and experienced. For example, a woman observes a midwife and a health visitor communicating about her care. Midwives’ and health visitors’ professional competencies as applied to care provision represent the *actual* level; these influence the empirical, and are not always observable [20]. At the deepest level – the *real* – are the generative mechanisms causing the observable events. For example, relational factors such as mutual trust for each other [22] and healthcare professionals’ limited control of financial or structural constraints imposed by the healthcare system [14]. Therefore, women’s experiences of collaborative care as provided by midwives and health visitors are key to better understanding their care needs and service provision more generally. This study aimed to explore women’s (i) experiences of maternity care as collaboratively provided by midwives and health visitors, and (ii) their perspectives of how their maternity care can best be provided by these healthcare professionals together.

## Methods

### Study design and setting

This study applied a qualitative, cross-sectional design, to elicit women’s views and experiences of midwife-health visitor collaboration. Focus groups were selected as they are an accessible, flexible method for generating data on a selected topic [23], and capitalise on group interaction [24]. Midwife-health visitor collaboration may be difficult to discuss for some women, due to limited experiences of this; thus, focus groups are appropriate because participants can comment on each other’s views and experiences, ask each other questions and seek clarification [23, 24]. Therefore, there is an immediate opportunity to compare and contrast experiences [24]. Data collection took place in a Children’s Centre in London, England.

### Participant recruitment

To be eligible to participate in this study, mothers needed to be over 18 years of age, have a child less than 18 months old, read and speak English and provide written consent to participate. Participant recruitment was approached through face-to-face contact with women in Children’s Centres, word of mouth, and social media (i.e. Twitter), enabling wide dissemination of study information.

A specifically developed topic guide was used (Additional file 1), informed by our systematic review of midwife-health visitor collaboration [9] and the research aims. As aligned with a critical realist approach, we presented a summary of the existing literature and sought women’s views of midwife-health visitor collaboration, and encouraged them to speak and discuss the topic freely and flexibly [20, 21]. Broadly, this covered: Experiences of women’s maternity care as provided by midwives and health visitors, opinions of health visitor antenatal contact, and women’s envisaged ideal maternity care pathway as collaboratively provided by midwives and health visitors.

### Data collection

In order to capture as diverse a population as reasonably possible, 74 Children’s Centres and other community-based groups in and around London were approached by email with a follow up email 1 week later. Of these, 10 centres responded (13.5%) to the email contact, with three agreeing to participate. Due to logistical constraints (e.g. Children’s Centre closures, lack of availability of co-moderator), face-to-face recruitment focussed on two Children’s Centres.

Following ethical approval from the Centre for Maternal and Child Health Research, School of Health Sciences (ref.: MCH/PR/PhD/17–18/01) in June 2017, recruitment commenced. Two members of the research team (RA, RB) were present at the focus group discussions. One acted as moderator (RA), and the other as assistant moderator (RB) responsible for note-taking. To accommodate women’s babies, baby bouncers and a soft play area was organised. All focus groups were audio-recorded following written informed consent of all participants.

The focus groups started with introductions, followed by sharing of experiences of meeting midwives and health visitors, and care women received. Participants were also invited to discuss their opinions on midwife-health visitor collaboration. Then, as a group, participants were invited to consider the maternity care pathway, and draw out their ideal collaborative maternity care model. Previous research has demonstrated that visual approaches can enhance discussion by representing relationships between the topics discussed, thereby increasing one’s understanding of a situation [25]. The aim of this exercise was to identify the most important aspects of maternity care that needed to be delivered collaboratively by midwives and health visitors. Including a visual approach to the focus group offered the opportunity to explore not only what women valued in relation to midwife-health visitor collaboration, but also construct their shared understanding of this beyond their individual experiences – a cornerstone of critical realism [20, 26]. Finally, participants were thanked for their participation in the study and offered a £10 voucher as a token of appreciation.

### Analysis

Audio-recordings were transcribed verbatim by a professional transcription agency and checked for accuracy by RA. Thematic analysis was applied to the data corpus in order to identify repeated patterns (i.e. themes) within the data, following several phases of analysis [27, 26]. Critical realism seeks to develop an understanding of reality through active engagement with existing knowledge and experience; as such, a combination of inductive and deductive thematic analysis was used [27], allowing for the developed themes to be guided by the data, the research aims, and the existing literature [9].

The first author led data analysis using QSR NVivo 11.4.1 [28]. The first steps were data driven [27], specifically: Familiarisation, or the reading and rereading of transcripts and generation of initial codes to identify noteworthy topics. Then, this was combined with a deductive approach: Codes were based on the research aims, and used as a template to organise the data into themes. This phase of the analysis was an iterative process and involved exploring the relationships between the themes and subthemes, and the research aims. A member of the research team (RB) reviewed the themes derived from the analysis, which involved assessing whether the codes within the themes were appropriate, and discussing these with the first author. Finally, a member of the research team (EO) read the themes (and codes within these) agreed upon by the two researchers (RA, RB) to ascertain whether these themes were representative of the data and made recommendations for defining and naming the final set of themes. One of the researchers (RB) is a midwife and health visitor, and two are health psychology researchers (RA, EO). The participants were not aware of the researchers’ clinical and academic backgrounds.

**Fig. 1:**
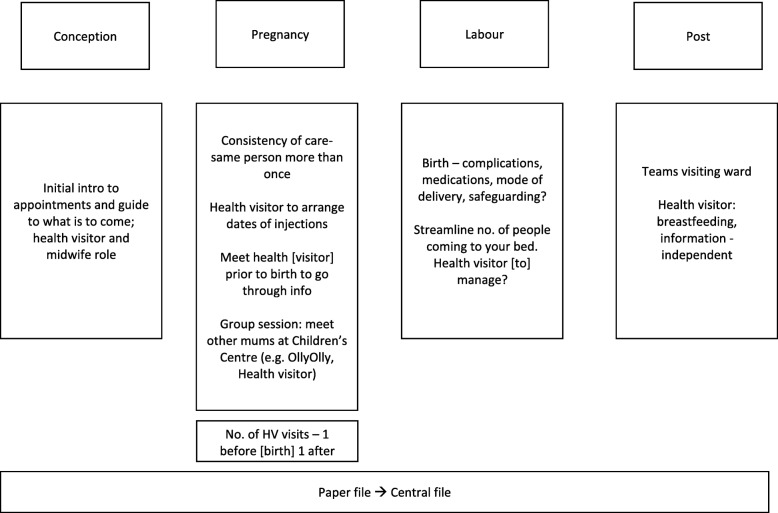
Focus group 1 transcribed group exercise notes outlining their ideal maternity care pathway

**Fig. 2:**
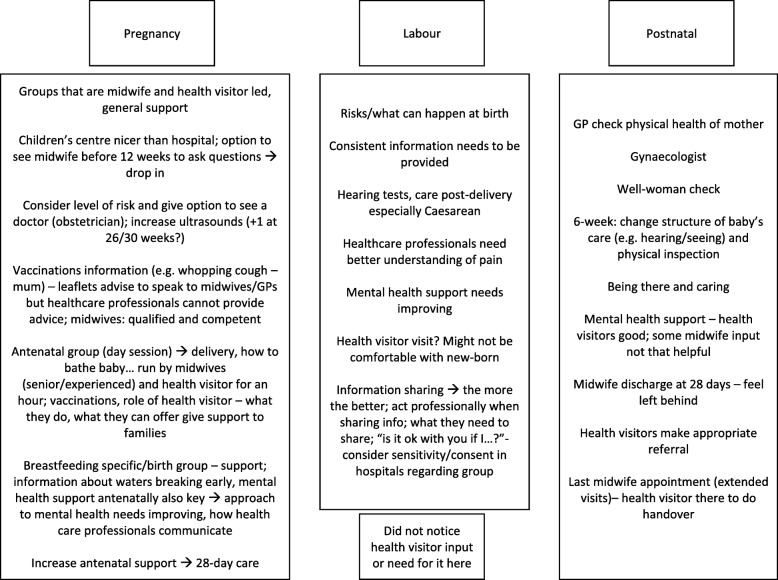
Focus group 2 transcribed group exercise notes outlining ideal maternity care pathway

**Fig. 3:**
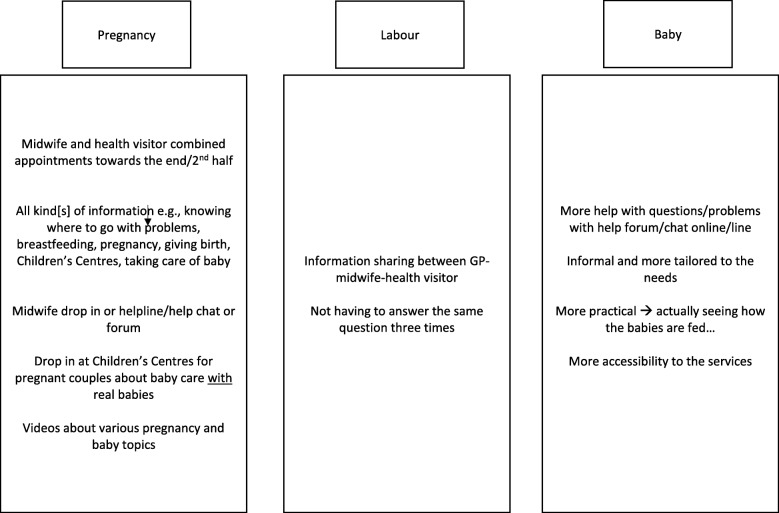
Focus group 3 transcribed exercise notes outlining ideal maternity care pathway

The data obtained from the group exercise concerning women’s descriptions of their ideal maternity care pathway were summarised narratively using notes on flipcharts and transcriptions (see Figs. 1, 2, and 3), and then compared and contrasted.

## Results

### Sample characteristics

Twenty-two women expressed interest in the study by providing their contact details. Twelve women participated (54.5%) across three focus groups. Reasons for dropout included ill health and schedule clashes. All bar one of the women had given birth to all their children in London. The remaining participant had given birth in London and another country outside of the UK. Participant characteristics are summarised in Table 1.

**Table 1:** Participant characteristics

Age range (mean)	30–44 years (34.67 years)
Ethnicity	
White English/British	3
White Other	5
Asian/Asian Other	2
Mixed (White and Black African/French; White and Black Caribbean)	2
Range of number of children	1–2 children
Age range of youngest child (mean)	1.75–8 months (5.06 months)

### Themes

Data analysis resulted in four themes, these are presented below (see Table 2 for a summary).

**Table 2:** Summary of themes linked to research aims

Research aim	Theme
To explore women’s experiences of maternity care as collaboratively provided by midwives and health visitors	Women’s experiences of maternity care from midwives and health visitors
To explore women’s perspectives of how their maternity care can best be provided by these healthcare professionals together.	Midwife-health visitor communication
	Midwife-health visitor collaboration for tailored care
	Women’s ideal maternity care pathway

#### Theme 1: Women’s experiences of maternity care from midwives and health visitors

This theme represents women’s relationships with midwives and health visitors, and their experiences of care as collaboratively provided by these healthcare professionals. Women’s accounts comprised of both positive and negative experiences of care, which related to their expectations of care from midwives and health visitors. Positive experiences were characterised by helpful midwives and health visitors, who provided them with extensive support. Women linked this to healthcare professionals’ experience and manner of communicating with them:

*I really liked our health visitor, she was really interested, she was very, she seemed professional at what she was doing, caring, like you said, I felt like she really sort of saw you as an individual not just like tick the box type of thing, and so that was really nice.*

Focus group (FG) 2, P7

Some women reported having less positive experiences from both healthcare professionals, such as pressure to breastfeed with limited support on how to achieve this. Women observed a lack of engagement from health visitors, particularly when going through routine questions:

*So she [a health visitor] just went through loads of forms and just circled stuff, you know, that I had to remember and then that was it really and then she went, but yeah, it was, it was a bit of a waste of time, really. […] There wasn’t much point to the visit.*

FG 1, P2

Although the majority of the participants reported not having any contact with their health visitors antenatally, those that did shared that this contact was positive, individualised and supportive:

*So I met my health visitor, contacted me when I was pregnant and met me […] and we just had an introduction […]. And I thought it was quite helpful actually, because it was quite nice that we’ve already got to know her then, and then she said that she would be my health visitor and be the person who’ll come and see us once the baby got home and she did.*

FG2, P10

Moreover, women expressed a desire to meet health visitors in pregnancy to establish rapport, process the information that is given to them, and ask questions about areas of care that they are concerned about (e.g. parent groups, immunisations):

P1: *[…] if they could introduce themselves to you before you have the baby, but …*

P3: *Yes, I definitely agree that to have the session that I had at home, to have had that prior to birth would have been much more helpful, because it [New Birth Visit] was literally leaflet after leaflet after leaflet, and then I keep meaning to go through it and you know, it takes a while to get round, […] some downtime so even prior, just before the birth, I would have found it more beneficial, just information overload I think at a really quite manic time.*

FG 1

#### Theme 2: Midwife-health visitor communication

Regarding midwife-health visitor communication, women reported that this appeared to take place largely via their Red Book^1^ [29] or notes, and observed evident fragmentation between midwives and health visitors:

*Mine were definitely completely fragmented, because on the days that […], but perhaps that was to do with the miscommunication initially with the addresses, but I would get a call from the health visitor on the day the midwife was coming, saying she was coming and I’d have to say no, I’ve already seen the midwife today, so there was definitely no communication between the teams in my experience.*

FG 1, P3

Although women reported limited communication between midwives and health visitors taking place, they noticed that midwives had an awareness of health visitors being responsible for seeing women at home postnatally: *“No, no, but the midwives did check if the health visitor was coming, so yeah, so I think there was some connection, but yeah that’s it really”* (FG 1, P1)*.*

In addition, women in this study reported valuing continuity of care, and of carer. Women shared experiences of seeing various health professionals throughout their pregnancy and after the birth, reflecting a lack of continuity of carer. This was associated with variations in the level and quality of care that the women receive as well as conflicting advice:

*…they [midwives and health visitors] all came to my house on the same day, after they got back they must have talked and I asked them one question about what kind […] oil to put on the baby because she was quite bad and they couldn’t agree. So they then had a disagreement about what the midwife was recommend this and the health visitor would recommend this, and they kind of knew that they would give different recommendations but, so they were talking amongst themselves and then I was like, oh I’ll just ask someone else.*

FG 3, P10

However, participants acknowledged that whilst it would be ideal to have a single healthcare professional providing maternity care (i.e., one named midwife and one named health visitor) this might not be feasible.

#### Theme 3: Midwife-health visitor collaboration for tailored care

This theme represents maternity care areas where midwife-health visitor collaboration could be beneficial. Participants focussed on information that they received from midwives and health visitors, and the co-ordination of their care.

Women expressed concerns regarding the inconsistent information they received and suggested that centralised records accessible to midwives and health visitors could be useful. All participants agreed it was important for midwives and health visitors to be aware of their health status and relevant medical information, to counter unnecessarily narrating their needs repeatedly. This aligns with women’s suggestions on how maternity care should be co-ordinated; in particular, through midwife-health visitor led antenatal group classes, and joint midwife-health visitor appointments. They considered group classes apt for learning about pregnancy and parenting from midwives and health visitors, along with other parents-to-be:

P2: *Even if they [midwives and health visitors] did a group session within the area, so you, it’s a chance to meet other mums going through the same thing before giving birth.*

*[…]*

P4: *Yeah, that’s true because things like what immunisations they need could be said.*

P2: *Yeah. And that gives a chance for mums to share any concerns and stuff at the same time and they’re all at the same stage of their pregnancy, so that’s nice.*

FG1

Participants acknowledged that there could be greater clarity around the health visitor’s role in order to maximise women’s engagement with them. This was especially important for women who immigrated to the UK. Women expressed interest in learning about what health visitors can offer, and midwives could help to facilitate this:

*[…] for me it would have been nice to meet them during their [midwives’] session and have them deliver something for 20 min or maybe on their role, what they do, da, da, da, da, that might be quite nice.*

FG 2, P5

Women suggested that joint appointments/visits could enable transfer of care particularly in the postnatal period: *“So maybe it would be helpful if they have a last midwife appointment, the health visitor would be there then to do a handover”* (FG 2, P7). In addition, participants identified Children’s Centres as potentially useful venues for meeting midwives and health visitors, along with other parents and/or parents-to-be, to do group activities and seek support and/or expert advice:

*Yes, actually being introduced to the children’s centre, I think lots of mums don’t actually get automatically told about children’s centres and playgroups and things like that, and actually it can be a real life saver in those first few weeks.*

FG 1, P1

#### Theme 4: Women’s ideal maternity care pathway

Drawing from the women’s accounts and visual data (see Figs. 1, 2 and 3) from the group exercise, this theme sets out participants’ suggestions for improving maternity care from pregnancy up to the postpartum period. It has been divided into pregnancy, labour/birth and postpartum care.

Table 3 summarises the common suggestions made by women across the three focus groups.

**Table 3:** Aggregated suggestions for improving the maternity care pathway as collaboratively provided by midwives and health visitors

Pregnancy	Labour/birth	Postnatal
• Midwife-health visitor combined session/appointment	•Information sharing between midwives and health visitors	•Increased postnatal support
◦ Content: Introduction session, guidance regarding what is to come, opportunity to ask questions	◦Ensure that information shared is consistent or accurate	◦Make appropriate referrals
◦ Format: Group or combined midwife-health visitor appointment	◦Obtain consent in a respectful way	◦Increase mental health, and breastfeeding support
◦ Where: Group sessions could be at Children’s Centres		
◦When: Towards end/second half of pregnancy		

##### Pregnancy

In pregnancy, all three groups agreed that a midwife and health visitor-led group session would be beneficial to guide parents-to-be about what is to come, and could be less labour intensive for midwives and health visitors. Regarding this session’s content, women wanted to be better informed about their maternity care pathway particularly the appointments they would be invited to, the health professionals leading each of these, and their respective role remits:

P5: *Yeah, I didn’t really know what they [health visitors] do really.*

P7: *I thought this was a general check that they do and I could imagine that for, they’re also maybe checking for everyone so that they can catch on families where things are difficult, but they are there to help, maybe. I thought that was part of their role, just to make, inform a little bit about, there’s a pathway of vaccinations to some general bits, but also to check if the baby is fine, where maybe families are difficult, that’s what I sort of thought was their role as well, just to check on everyone but if there’s some trouble that they could pick it up and help.*

FG 2

Women reported preferring a group setting, to meet other parents-to-be, and hear about questions or concerns other than their own. Sessions could be offered towards the end of pregnancy or in the second half of the pregnancy, to give women a chance to reflect on the information they are given:

P4: *I think so, but maybe not too early on, because, I don’t know about everyone else but when I was first pregnant especially I was just so wrapped up in the pregnancy I found it really hard to imagine actually having a baby and it felt faraway still, so maybe towards the end of the pregnancy when you’re like, OK, I do actually have to look beyond.*

P1: *And also I think towards the end of your pregnancy, you, like you were saying, you had a bit more time, some, not everyone, but some people have, will have a bit of, will stop work a bit before the baby’s due, and actually I think it would be quite nice if they did do a session in the children’s centre.*

FG 1

##### Labour/birth

There was consensus across the groups that birth-related and other relevant information (e.g. safeguarding issues) should be shared by healthcare professionals; midwives and health visitors having good knowledge of women’s history demonstrates connectedness between them. Participants were confident that the healthcare professionals involved in their care would share only pertinent information about women/families relevant to their welfare. At the same time, some women recognised that this could be more challenging for women who might present with vulnerabilities. Reflecting on personal experiences, however, the majority were comfortable with midwives and health visitors sharing information about them:

*[…] I do, personally, I’m happy with that information being shared between health visitor and midwife, I think it’s quite helpful and […] if the health visitor knows that you’ve had a C section, you’ve had a particularly traumatic birth, then they can be a bit more sensitive, I think that’s quite useful.*

FG 1, P1

Women were enthusiastic about centralised files that healthcare professionals could access in order to share accurate information with each other, and provide women with adequate, individualised support and advice:

P9:* […] if there could be something, like I said tongue tie it is in their system, I know it’s categorising and listing again, but if we make it a bit more focussed and individualised rather than give you general knowledge, then perhaps that is something that midwives and health visitors could easily share.*

FG 3

One participant (FG 1) had experience of meeting various healthcare professionals who had shared access to her information, which she appreciated.

##### Postpartum care

Participants’ key recommendation for postpartum care was increased support from midwives and health visitors. The groups had a variety of suggestions including having the opportunity for informal discussions with healthcare professionals (e.g. drop-ins), midwives and health visitors ensuring appropriate referrals, and providing adequate breastfeeding and mental health support:

P7: *I think what could be quite useful is if there was groups in general, maybe led by midwives or health visitors, during pregnancy, and afterwards for mums to just go to, so not just for breastfeeding, but say, you know you’re not feeling well afterwards.*

P6: *Yeah*

[…]

P5: *Like a support group almost.*

FG2

Extended midwife and health visitor involvement was important for making women feel cared for and supported, with their specific needs addressed.

## Discussion

This study explored women’s experiences of midwife-health visitor collaboration, as well as their ideal maternity care pathway. The main findings are: 1) women’s experiences of maternity care as delivered by midwives and health visitors are varied, 2) women perceived the communication between midwives and health visitors as limited, fragmented, and associated with conflicting advice, and 3) collaboration throughout the maternity pathway could be beneficial particularly in relation to information-giving and care co-ordination. Each of these will be discussed sequentially.

First, concerning midwife-health visitor collaboration, the findings showed that women’s experiences were a mixture of positive and negative ones, supporting previous research [30, 31]. For example, a national survey of women’s maternity care in the UK (N= > 4500) found that over 75% of the respondents had positive care experiences [32]. Women in this study reported valuing their relationships with midwives and health visitors who are supportive (e.g. showing interest in mother and baby) and active listeners. Interestingly, despite the women living in the same geographical area with similar service providers, women still reported varied experiences of care particularly in relation to contacts with health visitors in pregnancy. One possible explanation is the decline of the health visiting workforce in England by 10% (9491 vs. 8588 Full-Time Equivalent) in the 12-month period between June 2016–2017 [33]. In addition, there has been a 16.88% reduction of antenatal contacts carried out nationally in Quarter 2 of 2017 (60,853 contacts) compared to Quarter 2 of 2016 (73,213 contacts) [34]. A critical realist approach would also suggest that factors at the real level (e.g. healthcare workforce structure) are influencing the *empirical* level given the diversity in women’s reported experiences [18–21].

Second, women reported observing service fragmentation, evidenced by scant communication between midwives and health visitors. This reflects findings from the National Maternity Review, where women emphasised that good communication and information sharing amongst health professionals is essential [35]. However, participants also acknowledged that women’s needs differ, which could partially explain why they reported limited midwife-health visitor communication. Research has shown that shared goals (e.g. smoking cessation targets) enhanced relationships between healthcare professionals including midwives and health visitors, which could be directly observed by women who are in contact with such services [36]. Women reported continuity of care, and of carer as important. This has been shown to be linked with lower mortality in terms of continuity with doctors [16] and needs to be explored in the context midwifery and health visiting. Whilst NHS England [17] also highlights the value of continuity of care, and of carer, this is focussed on midwifery care.

Finally, women contributed strategies for improving current maternity care provisions, aligned with the National Maternity Review [35] and Public Health England and Department of Health (UK) midwife-health visitor partnership pathway [8], specifically focussed on interprofessional collaboration. These included service changes, most notably an increased offering of group-based antenatal care collaboratively delivered by midwives and health visitors within community-based services. Existing maternity care pathways set out in line with policies such as the *Healthy Child Programme* [1] recommend group-based antenatal classes delivered in community or healthcare settings to enhance social support. Accordingly, women in this study considered such classes as a valuable resource, and a channel through for obtaining social support. However, there is evidence to suggest that health visitor involvement in antenatal classes is lacking [36]. Thus, currently available classes [36] do not meet these women’s suggestion of classes jointly provided by midwives and health visitors and needs to be considered. Successful collaborative working in maternal health have been characterised by the provision of opportunities for health professionals to interact with each other and have shared activities [32], which was also reported to be influential by midwives and health visitors. Taken together, the evidence highlights the potential value of group antenatal classes for women, midwives, and health visitors alike.

The participants also made recommendations for improving mental health and breastfeeding support. Both are core requirements of delivering the *Healthy Child Programme* [1]. Redshaw and Henderson’s [32] research showed that the majority of the women they surveyed (N= > 4500, 82%) reported having been asked about their mental health, mostly by midwives, in pregnancy. Similarly, 90% of those surveyed were asked about their mental health postnatally, with 63% of these women reported having received support [32]. It was not clear, however, which health professionals were involved in offering postnatal mental health support. It has been shown that group-based breastfeeding support interventions provided jointly by midwives and health visitors can improve breastfeeding, particularly when relationships between these healthcare professionals are strong [7]. One plausible explanation for the participants’ desire for increased breastfeeding and mental health support is the nature of their personal circumstances. For example, some participants reported having limited proximal familial/social support. However, this finding needs to be interpreted with caution in keeping with a critical realist approach because women will have different constructions and interpretations of reality shaped by the resources and/or support available to them (*actual* level), as well as their views on the kind of care that healthcare professionals ought to provide (*real* level). Despite these differences which the participants were aware of, they still had shared experiences and needs, suggesting that these layers only provide a partial understanding of the complex nature of reality [20, 21].

Finally, women suggested that their care pathway could be made clearer to them. This is in line with previous research, where women have stressed the value of being better informed about what they could expect from perinatal care such as the frequency of appointments and the purpose of these [30]. Generally, it is known that communication is paramount to high-quality maternity care, both from women’s/families’ and health professionals’ perspectives [35]. Communication was also reported as playing a pivotal role in enabling midwife-health visitor interprofessional collaboration [9], and identified by women as a key issue in maternal and child health [36]. Specifically, participants were confident in healthcare professionals’ ability to communicate information in an appropriate manner. They stressed that obtaining their consent for health professionals to share information with each other needs to be done in a respectful way. From a critical realist standpoint, this finding suggests that women lack an awareness of the barriers to communication experienced by midwives and health visitors, which are beyond these individuals’ direct/observed experiences. Specifically, these healthcare professionals do not presently have shared information systems [35], despite the evidence suggesting that such systems can facilitate collaborative working between these healthcare professionals [9, 10].

### Strengths and limitations of the study

A key strength of this study lies in the manner in which women’s views were elicited – through semi-structured interview questions, and women-led, open group discussion to visualise their ideal maternity care pathway [24]. This format allowed women to explore their experiences together, and comment on each other’s views and experiences. The diversity of the views obtained from the participants is a further strength of this study. In addition, as is recommended in focus group literature [23], the groups maintained a level of homogeneity in that they were all based in the same geographical area, with some women attending the same General Practice (GP) surgery. Furthermore, the participants were similar in terms of the number of children they had and gave birth in similar settings.

However, the participants were a self-selected sample, and evidently proactive about their maternity care (e.g. accessing Children’s centres that they advocated for). Additionally, there were pre-existing relationships between a few of the participants (i.e. some were known to each other) which could have influenced how they responded to the questions. However, all the women appeared comfortable in the group setting, and still openly discussed their experiences with the rest of the group.

### Clinical practice and research implications

The present study contributes to the body of knowledge by validating past research [35, 36], and enhances our understanding of maternity care collaboratively provided by midwives and health visitors from the recipients’ perspective. The findings indicate that it is paramount that women are listened to, offered consistent services, and provided unbiased information and advice by midwives **and** health visitors. In addition, women’s care pathways need to be made clear to them at the outset, including information about the health professionals who may be involved in their care, and these professionals’ roles. In terms of midwife-health visitor interprofessional collaboration, the participants showed an awareness of the issues previously raised by these healthcare professionals [10, 37–39] such as poor communication and limited access to shared information, thereby supporting the evidence on the identified barriers and enablers to collaborative working [9]. Whilst the recommendations presented here (e.g. group-based antenatal and postnatal appointments/drop-ins, centralised records) may not necessarily apply to nor be desired by all women, the findings highlight the importance of providing individualised care delivered collaboratively by midwives and health visitors. Thus, it is crucial that women’s voices are heard and considered when providing care [12, 35], ultimately promoting informed choice.

Considerations for future research include exploring specific service changes for improving maternity care pathways such as the feasibility of group-based antenatal classes jointly provided by midwives and health visitors, and evaluating the impact of midwife-health visitor communication on health (e.g. rates of postnatal depression, mortality) and service outcomes (e.g. referral management). Maternity care models and guidance developed should include health visitor input if services are to achieve midwife-health visitor collaboration throughout the care pathway. In addition, future research should include other stakeholders such as policymakers and service commissioners to obtain a better understanding of how midwifery and health visiting services could be redesigned to support collaborative working.

## Conclusions

This study explored women’s views and experiences of collaboratively provided maternity care by midwives and health visitors. Women reported limited midwife-health visitor collaboration; however, they acknowledged the potential value of collaborative working between these groups. Reflecting upon their experiences of care, women were able to identify the issues that they perceived could benefit from collaborative working, such as the provision of inconsistent or inaccurate advice, as well as fragmentation between services. Moreover, the participants offered potential solutions to these, such as the provision of group-based midwife-health visitor antenatal appointments. Women also highlighted positive experiences of the care that they received, such as having helpful midwives and health visitors. Women’s recommended strategies regarding how midwife-health visitor interprofessional collaborative practice could be improved demonstrate the necessity of their input in service development efforts.

## Additional files

Additional file 1: Focus group topic guide. This document outlines the topic guide used in the focus groups with women. (DOCX 105 kb)

## Abbreviations

GP: General Practice
NHS: National Health Service

## Footnotes

1 Red books, or Personal Child Health Records are standardised records of a child’s development provided to parents.
