# Supplementary material for: A focus group study of women’s views and experiences of maternity care as delivered collaboratively by midwives and health visitors in England
Source: BMC Pregnancy Childbirth. 2018 Dec 27;18:505. doi: 10.1186/s12884-018-2127-0 (PMC6307134; doi:10.1186/s12884-018-2127-0)
Supplement: Supplementary file 1 — Focus group topic guide. This document outlines the topic guide used in the focus groups with women. (DOCX 105 kb) [file 12884_2018_2127_MOESM1_ESM.docx]

**Focus group topic guide**

Introduction (10 minutes)

- Introduction to the study
- Consent (including audio recording)
- Demographic questionnaire
- Reminder about having no right or wrong answers – interested in what your views are on ideal care pathway provided by midwives and health visitors based on your experiences

Ice breaker (5 minutes)

- How did you hear about the focus group?
- Tell us about when you met your health visitors – antenatally? If no, would you have liked to have met them?

Current evidence (approx. 20 minutes)

- Summary of what we know about how midwives and health visitors work together
- Your views (prompts: What are your views on this? Is there anything that midwives/health visitors haven’t identified in terms of what you think might encourage them or stop them from working together? If so, what?)

Designing your ideal care pathway (approx. 25 minutes)

- In small groups, write out your ideal care pathway from pregnancy until 6 weeks after the birth (prompts: Based on your previous experience, how many contacts would you like to have had from midwives and health visitors? What information should be passed on, when, and how? Why are these important to you? To what extent would you like to be involved in how your care is co-ordinated, and why? What can be improved in terms of how midwives and health visitors provide their care?)

Break (approx. 15 minutes)

Consensus discussion (30 minutes)

- Discuss each group’s pathway (prompts: Were there any contact points identified by the groups that were missed, or that are unnecessary? What are your views on the means of communication and information sharing between you and the midwives and health visitors involved?)
  - Identify common themes and those that stand out/contrasting points

Closing and thanks (15 minutes)

- Hand out token of appreciation
